# Contrast Sensitivity Is Associated With Chorioretinal Thickness and Vascular Density of Eyes in Simple Early-Stage High Myopia

**DOI:** 10.3389/fmed.2022.847817

**Published:** 2022-03-24

**Authors:** Xinting Liu, Yanli Wang, Xiaoxiao Ying, Fen Zhang, Jing Huang, Hang Yu, Qian Wang, Miaoran Zheng, Fang Hou, Luis Lesmes, Zhong-Lin Lu, Fan Lu, Xinjie Mao

**Affiliations:** ^1^School of Ophthalmology & Optometry and Eye Hospital, Wenzhou Medical University, Wenzhou, China; ^2^Cixi People’s Hospital, Cixi, China; ^3^Adaptive Sensory Technology, Inc., San Diego, CA, United States; ^4^Division of Arts and Sciences, NYU Shanghai, Shanghai, China; ^5^Center for Neural Science, Department of Psychology, New York University, New York, NY, United States; ^6^NYU-ECNU Institute of Brain and Cognitive Science, NYU Shanghai, Shanghai, China

**Keywords:** simple high myopia, contrast sensitivity, qCSF, choroidal thickness, retinal thickness and vascular density

## Abstract

**Objective:**

To evaluate the contrast sensitivity function (CSF), chorioretinal thickness and vascular density as well as their relationships in subjects with simple early-stage high myopia.

**Methods:**

Eighty-one young subjects were enrolled in this study. They were categorized into the simple high myopia group (sHM, *n* = 51) and the low-moderate myopia group (control group, *n* = 30). Monocular CSF under best correction was measured with the qCSF method. Retinal superficial and deep vascular density, inner and outer retinal thickness and choroidal thickness were measured using optical coherence tomography angiography.

**Results:**

The area under log CSF (AULCSF) and cutoff spatial frequency (Cutoff SF) of the sHM group were significantly reduced compared to those of the control group (*P* = 0.003 and *P* < 0.001, respectively). The parafoveal and perifoveal retinal thickness, deep vascular density and choroidal thickness were also significantly reduced in the sHM group (all *P* < 0.05). Multiple regression analysis revealed that AULCSF was significantly correlated with retinal deep vascular density, outer retinal thickness in the parafoveal and perifoveal areas (all *P* < 0.05).

**Conclusion:**

Compared to low to moderate myopic eyes, patients with simple high myopia have thinner retinal and choroidal thickness, lower retinal vascular density, and reduced contrast sensitivity. Moreover, the CSF was correlated with the measures of chorioretinal structure and vasculature. The results suggest that the CSF is a sensitive functional endpoint in simple early-stage high myopia.

## Introduction

High myopia is one of the most common causes of visual impairment in the world, especially in Asia. It is estimated that by 2050, the number of people with high myopia in China will exceed 175 million, accounting for 13% of the total population (1). Complications related to high myopia, such as maculopathy, choroidal neovascularization, and retinal detachment, are the main causes of severe visual impairment and blindness. Thus pathological myopia has been recognized as a leading cause of blindness (2). However, the natural development of pathological myopia is slow. Traditional functional endpoints, such as the best-corrected visual acuity, are usually not affected until the late stage of the disease when significant pathological changes have occurred (3). Therefore, a more sensitive functional endpoint is needed to diagnose and guide the treatment of pathologic myopia in the early stage.

The criteria for simple high myopia (sHM) are spherical equivalent refractive error ≥−6.0 D that stabilizes in adulthood, and no other ocular pathology. Many studies have shown that the thickness of the choroid and retina in simple high myopic eyes are reduced (4–6). In addition, Ye et al. (6) have reported that vascular density of the retina in simple high myopic eyes are also lower than those in emmetropic and low-to-moderate myopic eyes (6). With full optical correction, retinal structure is the earliest limiting factor that could affect visual function in the visual pathway. Changes in the retinal or choroidal vascular bed can lead to impaired visual function (7).

The contrast sensitivity function (CSF) describes contrast threshold in a wide range of spatial frequencies (8). It is a more comprehensive and sensitive endpoint for functional vision than visual acuity and is better correlated with daily visual functions (9, 10). It has been reported that patients with simple high myopia had reduced CSF compared to people with normal vision (10, 11), yet the cause of the CSF deficits remains unclear. In addition, CSF has been used to evaluate retinal function in many studies (12–16). We hypothesized that decreased contrast sensitivity is associated with structural changes of the fundus in patients with simple high myopia.

Traditional laboratory CSF tests are not suitable for clinical practice because they take a long time to administer (about 30–60 min). In response to this challenge, Lesmes et al. developed a novel Bayesian adaptive psychophysical procedure, the qCSF method (17), that was further improved by incorporating a 10-alternative forced-choice (10AFC) identification task (18, 19). The new qCSF test can provide a highly precise and accurate CSF assessment in 3–5 min (18, 19).

In this study, we applied the qCSF procedure to assess CSF in subjects with simple early-stage high myopia and used optical coherence tomography angiography (OCTA) to measure retinal vascular density, retinal thickness, and choroidal thickness in foveal (1 mm), parafoveal (1–3 mm), and perifoveal (3–6 mm). The relationship between CSF metrics and measures of fundus microstructure was also evaluated.

## Materials and Methods

### Subjects

All subjects for this cross-sectional study were recruited from the Eye Hospital of Wenzhou Medical University between December 2019 and July 2020. The study protocol adhered to the tenets of the Declaration of Helsinki and was approved by the Human subjects’ Research Review Board of the Eye Hospital of Wenzhou Medical University. Written informed consent was obtained from each subject before the experiment. The study protocol was registered in Chinese Clinical Trials Registry (Registration No. ChiCTR2000040926).

In our study, the participants were all examined by two of the co-authors (XL and YW), who are certificated ophthalmologists, following standard procedure. All subjects underwent comprehensive ophthalmologic examinations, including manifest refraction, best-corrected distance visual acuity (BCDVA) and log MAR acuity assessment, and slit lamp biomicroscope. Axial length (AL) was measured by optical low-coherence reflectometry (LENSAR, LS 900, SN 1694, V1.1.1), non-contact IOP was measured by a Full Auto Tonometer TX-F (Topcon, Tokyo, Japan), and fundus photography was taken with Hybrid Digital Mydriatic Retinal Camera CX-1 (Canon Inc., Tokyo, Japan).

The subjects were divided into two groups according to their spherical equivalent (SE) correction: the control group with SE ranging from +0.50 to –6.0 diopters (D), and the simple high myopic (sHM) group with SE ≥−6.0 D. All participants had normal or corrected-to-normal vision (≥20/20), astigmatism not greater than 1.50 D, interocular difference of refractive error less than 1.00 D, and no eye disease other than refractive error. Subjects with less than 20/20 BCDVA in either eye, intraocular pressure (IOP) more than 21 mmHg, visual field defects, history of intraocular surgery, complications of high myopia such as retinoschisis or choroidal neovascularization, systemic diseases, or used systemic or topical medications that may affect accommodation or binocular vision, were excluded. All tests were conducted with the right eye.

### Optical Coherence Tomography Angiography Measurements

**FIGURE 1:**
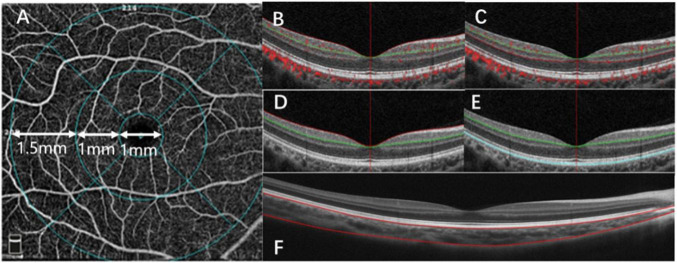
Optical coherence tomography angiography (OCTA) image analysis. **(A)** A fundus image, with the macula divided into three subfields: the fovea (1 mm), parafovea (1–3 mm), and the perifovea (3–6 mm). **(B)** OCTA image of the retinal superficial vascular density (SVD). **(C)** OCTA image of the retinal deep vascular density (DVD). **(D)** OCTA image of the inner retinal thickness (IRT). **(E)** OCTA image of the outer retinal thickness (ORT). **(F)** OCTA image of the choroidal thickness (ChT) in macular.

All subjects were thoroughly examined using OCTA (Optovue RTVue XR Avanti; Optovue, Inc., Fremont, CA, United States). The RTVue OCT scanning speed was 70,000 A-scans per second, and the wavelength of the light source was 840 nm with a 50 nm bandwidth. Each OCTA image was composed of 304 pixels in the horizontal and vertical directions. A greater than 50 signal strength index indicates that the center of the scan is well aligned and the image can be used for further analysis (20). Choroidal thickness was measured using the device’s unique deep choroidal imaging (DCI) mode to perform horizontal and vertical cross-scans (Figure 1F). Horizontal and vertical images were used for choroidal thickness measurement. To reduce the influence of diurnal variation on the choroid and ensure consistency and accuracy of the results, all measurements were conducted by the same physician between 13:30 and 17:00 (21).

Bennett’s formula was used to adjust ocular magnification, and all images were corrected using axial length (AL). The actual scan diameter t was determined by the formula *t* = p × q × s, where p represents the magnification factor of the camera of the OCT imaging system, *q* = 0.01306 × (AL−1.82) (22), represents the magnification factor in relation to the eye, and s represents the original measurement value obtained from the OCT image.

The OCTA scan area was centered on the fovea with a 6 mm × 6 mm field of view, which corresponded to 10°. We divided the macular into three areas, the fovea, parafovea, and perifovea. The fovea was defined as a 1-mm diameter disk around the center of the macula. The parafovea was defined as an annulus with a 1 mm inner diameter and a 3 mm outer diameter, centered on the center of the macula. The perifovea was defined as an annulus with a 3 mm inner diameter and a 6 mm outer diameter, centered on the center of the macula (Figure 1A).

The AngioVue software can be used in several layouts with multiple retinal layer segmentations. The OCTA system automatically provided the average inner and outer retina thicknesses. The layers between the inner limiting membrane (ILM) and the outer boundary of the inner plexiform layer (IPL) was defined as the inner retina (Figure 1D), and the layers between the outer boundary of the IPL and the RPE-Bruch membrane complex was defined as the outer retina (Figure 1E). Automatic segmentation was performed by the visualization software to generate en-face projections of the superficial vascular density (SVD) and deep vascular density (DVD) of the retina. Automatic segmentation was performed with The Split-Spectrum Amplitude-Decor- relation Angiography (SSADA) algorithm to generate enface projections of the superficial vascular density (SVD) and deep vascular density (DVD) of the retina. The SSADA technique has several potential advantages over phase-based techniques, including insensitivity to phase noise and the ability to quantify microvascular flow. The algorithm is very precise and has been used to segment the vessels (23). The SVD extended from the ILM to 9 μm above the IPL (Figure 1B). The DVD extended from 9 μm above the IPL to 9 μm below the outer plexiform layer (OPL) (Figure 1C). The choroidal thickness was extracted and measured by customized logarithms using MATLAB R2017a with AL correction. Segmentations of RPE–Bruch’s membrane complex and the choroid-sclera interfaces were adjusted manually by a trained examiner.

The following parameters were evaluated: retinal superficial vascular density (SVD), retinal deep vascular density (DVD), inner retinal thickness (IRT), outer retinal thickness (ORT), and choroidal thickness (ChT).

### The Quick Contrast Sensitivity Function Method

**FIGURE 2:**
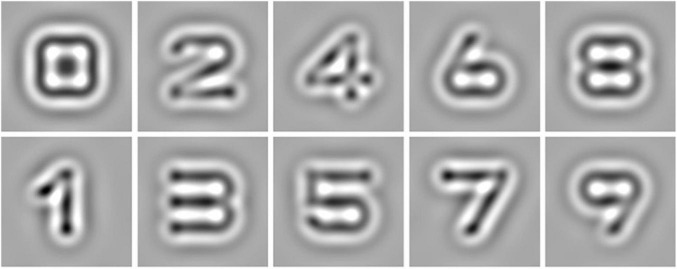
Band-pass filtered digits used in the qCSF test.

The 10-digit qCSF method (19) was implemented in MATLAB (MathWorks, Natick, MA, United States) with Psychtoolbox extensions and run on a Mac minicomputer (Model No. A1347; Apple, Inc., Cupertino, CA, United States). The stimuli were displayed on a gamma-corrected Asus flat panel monitor (PG279Q; Asus Corp., Taipei, Taiwan) with a background luminance of 91.2 cd/m^2^. The spatial resolution of the display was 2560 × 1440 pixels and the refresh rate was 60 Hz (19). At a viewing distance of 1.34 m, each pixel suspended 0.018°. A bit-stealing algorithm was used to achieve 9-bit gray-scale resolution (Figure 2) (24). Observers viewed the display with their right eye under the best correction, if any, in a dark room. The left eye was patched during the test.

All subjects viewed the test stimuli with the best refractive correction during the CSF test. Before the test, each subject had 5 min to adapt to the dark test environment. In the beginning of the test session, each subject was given a few practice trials to familiarize themselves with the experimental settings and procedures. In each trial, a short tone indicated its beginning, and a fixed crosshair (250 ms) was displayed in the center of the screen, followed by a blank screen (125 ms) with background luminance. Then, a filtered digit stimulus was presented for 133 ms, followed by a 500 ms response screen. The digits on the response screen were arranged in a 2 × 5 matrix and presented in the center of the display. Subjects used the computer keyboard to select the digit they saw. No feedback was provided to the subject during the experiment. A new trial started 500 ms after the response.

To characterize CSF differences between the study groups, the area under log CSF (AULCSF), which is a broad measure of spatial vision (14), and the cutoff spatial frequency (Cutoff SF), which characterizes the high-frequency resolution of the visual system, were calculated for each subject (25).

### Statistical Analyses

All data are presented as mean ± standard deviation and were analyzed with SPSS (version 26.0; SPSS, Inc., Chicago, IL, United States). Data were first tested for normality using the Shapiro–Wilk test. Independent-samples *t*-tests were used to evaluate the differences between the two groups. Pearson’s correlation, and linear regression analysis were used to evaluate potential relationships between CSF metrics and the thickness and vascular density of the retina and choroid. *P* < 0.05 was considered statistically significant.

## Results

### Basic Patient Characteristics

A total of 81 subjects (aged from 18 to 30 years), with 30 in the control group and 51 in the HM group, completed the experiment. The best-corrected visual acuity of the right eye of all the 81 subjects was 20/20 or better. The mean SE was −8.27 ± 0.21 D in the sHM group, which was more myopic than the SE (−1.70 ± 0.17 D) in the control eyes (*p* < 0.001). The mean AL was 26.99 ± 0.14 mm in the sHM group, much longer than that in the control group (24.47 ± 0.14 mm, *p* < 0.001). There were no significant gender and age differences between the two groups (*P* = 0.849 and 0.409, respectively; Table 1). The demographic and clinical information of the two groups is summarized in Table 1.

**TABLE 1:** Basic characteristics of the subjects.

	Control	sHM	*P*
Eyes	30	51	
Gender	13/17	21/30	0.849
Age (year)	23.67 ± 0.48	23.10 ± 0.44	0.409
SE (D)	−1.70 ± 0.95	−8.27 ± 1.49	**<0.001**
AL (mm)	24.47 ± 0.99	26.99 ± 0.98	**<0.001**

*Significant P-values are indicated in bold.*

### Chorioretinal Structure and Vasculature

The retinal SVD of the sHM group was significantly lower than that of the control group in parafovea (*P* = 0.041, Table 2). There was no significant difference between the two groups in the fovea and perifovea (Table 2). The retinal DVD of the sHM group was significantly lower than that of the control group in the parafovea and perifovea (both *P* < 0.001). There was no significant difference between the two groups in the fovea (Table 2).

**TABLE 2:** Retinal vascular density in the sHM and control groups.

	Control (95% CI)	sHM (95% CI)	*P*
**SVD (%)**			
Foveal	19.15 ± 6.78 (16.62–21.68)	21.13 ± 6.48 (19.31–22.96)	0.195
Parafoveal	52.14 ± 4.00 (50.65–53.64)	49.97 ± 4.83 (48.61–51.33)	**0.041**
Perifoveal	50.45 ± 3.28 (49.23–51.68)	49.56 ± 3.59 (48.55–50.57)	0.268
**DVD (%)**			
Foveal	36.39 ± 8.66 (33.16–39.63)	38.56 ± 7.66 (36.41–40.72)	0.245
Parafoveal	56.84 ± 3.55 (55.51–58.16)	52.57 ± 5.21 (51.10–54.03)	**<0.001**
Perifoveal	53.51 ± 4.78 (51.72–55.29)	46.81 ± 6.78 (44.90–48.72)	**<0.001**

*Significant P-values are indicated in bold.*

The retina and choroidal thickness of the two groups are listed in Table 3. Compared with the control group, the IRT of the sHM group was thicker in the fovea (*P* = 0.017) but was not significantly different in the parafovea and perifovea (Table 3). The ORT of the HM group was significantly thinner than that of the control group in the parafovea and perifovea (*P* = 0.003, *P* < 0.001, respectively), but there was no significant difference between the two groups in the fovea (*P* = 0.101). In addition, the ChT of the sHM group was significantly thinner than that of the control group in the fovea, parafovea, and perifoveal (all *P* < 0.001, Table 3).

**TABLE 3:** The retinal and choroidal thickness in the sHM and control groups (μm).

	Control (95% CI)	sHM (95% CI)	*P*
**IRT (μm)**			
Foveal	51.50 ± 8.81 (48.21–54.79)	56.29 ± 8.34 (53.95–58.64)	**0.017**
Parafoveal	111.04 ± 5.79 (108.88–113.20)	111.03 ± 5.92 (109.36–112.69)	0.993
Perifoveal	102.63 ± 6.44 (100.22–105.03)	100.03 ± 6.59 (98.18–101.88)	0.088
**ORT (μm)**			
Foveal	203.13 ± 11.45 (198.86–207.41)	206.90 ± 8.84 (204.42–209.39)	0.101
Parafoveal	211.93 ± 8.80 (208.65–215.22)	206.21 ± 7.82 (204.01–208.41)	**0.003**
Perifoveal	184.99 ± 7.57 (182.16–187.82)	177.33 ± 7.18 (175.31–179.35)	**<0.001**
**ChT (μm)**			
Foveal	277.68 ± 50.31 (258.90–296.47)	183.78 ± 76.11 (164.91–202.65)	**<0.001**
Parafoveal	276.29 ± 49.73 (257.72–294.86)	183.91 ± 63.72 (165.99–201.83)	**<0.001**
Perifoveal	265.36 ± 42.26 (249.58–281.14)	183.93 ± 60.08 (167.03–200.83)	**<0.001**

*Significant P-values are indicated in bold.*

### Contrast Sensitivity Function

**FIGURE 3:**
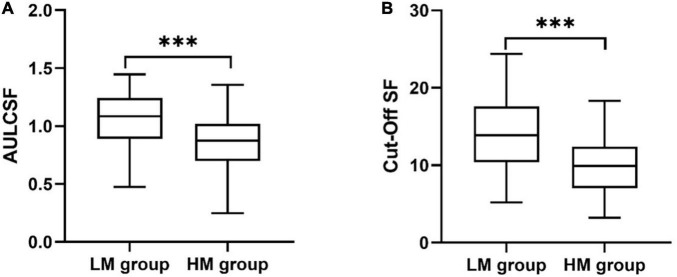
Metrics of the contrast sensitivity function for the sHM and control groups. **(A)** Area under the log CSF (AULCSF). **(B)** Cutoff Spatial frequency (Cutoff SF). ***P < 0.001.

As shown in Figure 3, the area under log CSF (AULCSF) of the sHM group was significantly less than that of the control group (0.85 ± 0.32 vs. 1.07 ± 0.46; *P* < 0.001). The Cutoff SF of the sHM group was 9.93 ± 0.47 cpd, which was significantly lower than that of the control group, 14.38 ± 0.98 cpd (*P* < 0.001).

### Relationships Between Contrast Sensitivity Function and Fundus Microstructures

Across all study eyes, the AULCSF and Cutoff SF were positively correlated with SVD, DVD, and ORT in the parafoveal and perifoveal areas (*r*-values ranged from 0.253 to 0.385, all *P* < 0.05, Table 4), and with ChT in all areas (*r*-values ranged from 0.290 to 0.352, *P*-values ranged from 0.001 to 0.009, Table 4). The AULCSF and Cutoff SF were not correlated with FAZ (*r* = 0.019 and −0.010, *P* = 0.864 and 0.930, respectively) or the IRT (*r* ranged from 0.057 to 0.102, *P* ranged from 0.365 to 0.612).

**TABLE 4:** Correlation between the contrast sensitivity function and the chorioretinal structure and vasculature in myopia.

	AULCSF		Cutoff SF	
	*r*	*P*	*r*	*P*
**SVD**				
Foveal	0.058	0.606	0.054	0.631
Parafoveal	0.330	**0.003**	0.350	**0.001**
Perifoveal	0.283	**0.010**	0.277	**0.012**
**DVD**				
Foveal	0.060	0.592	0.068	0.548
Parafoveal	0.272	**0.014**	0.296	**0.007**
Perifoveal	0.359	**0.001**	0.385	**<0.001**
**IRT**				
Foveal	−0.085	0.450	−0.062	0.580
parafoveal	0.101	0.372	0.102	0.365
Perifoveal	0.057	0.612	0.061	0.591
**ORT**				
Foveal	0.043	0.706	0.048	0.671
Parafoveal	0.301	**0.006**	0.309	**0.005**
Perifoveal	0.253	**0.023**	0.299	**0.007**
**ChT**				
Foveal	0.299	**0.007**	0.352	**0.001**
Parafoveal	0.297	**0.007**	0.351	**0.001**
Perifoveal	0.290	**0.009**	0.341	**0.002**

*Significant P-values are indicated in bold.*

**FIGURE 4:**
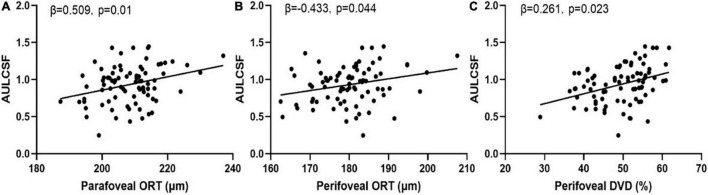
Scatter plots of measures of chorioretinal structure and vasculature vs. AULCSF. **(A)** Parafoveal outer retinal thickness vs. AULCSF; **(B)** Perifoveal outer retinal thickness vs. AULCSF; **(C)** Perifoveal deep vascular density vs. AULCSF.

The parameters that were significantly correlated with the AULCSF and Cutoff SF were included in the multiple regression analysis. Only outer retina thickness in the parafoveal and perifoveal areas and deep vascular density remained significantly correlated with AULCSF (Figure 4).

## Discussion

In this study, we measured the CSF and fundus microstructure of eyes with simple high and low to moderate myopia using qCSF and OCTA. We also evaluated the relationship between the CSF and measures of the fundus microstructure. We found that, in the fovea, IRT of the high myopia group was greater than that of the low-to-moderate myopia group, while there was no significant ORT difference between the two groups. However, in the parafoveal and perifoveal regions, ORT of the high myopia group was reduced compared to the low-to-moderate myopia group. Consistent with other studies (26, 27), the results suggest that patients with high myopia tend to have thinner peripheral but thicker foveal retina. This may be due to the stretching of the eyeball by the increased axial length in high myopia, which flattens the inner limiting membrane (28).

In addition to retina thickness, we found that the DVD of the parafoveal and perifoveal regions was significantly reduced in the high myopia group compared to that of the low-to-moderate myopia group, while there was no significant change of vascular density in the fovea, consistent with Al-Sheikh et al. (29) and Mo et al. (30). In fact, Milani et al. (31) has speculated that there may be a protective mechanism for the fovea in high myopia. We also found there was no significant SVD difference between the two groups except in parafovea. Previous studies have shown that the choroidal thickness of highly myopic eyes was significantly thinner than that of normal eyes or those with low-to-moderate myopia (32, 33). Furthermore, we found that the ChT of young patients with high myopia was significantly reduced. This is consistent with previous studies (32, 33).

High myopia, characterized by excessive and progressive elongation of the globe and mechanical stretching of the sclera at the equatorial region, can cause atrophy and degeneration in the retina and choroid, resulting in various secondary fundus diseases, and ultimately lead to abnormal visual function or even vision loss (2). Currently, the primary endpoint for functional vision is visual acuity measured with high contrast optotypes (3). Moreover, in clinical practice, we find that many high myopic patients complain about poor vision, even though they exhibit normal 20/20 letter acuity. The qCSF test assesses functional vision with optotypes with a wide range of sizes and contrasts. It is much more sensitive than the traditional VA test and can detect “hidden” vision losses when VA appears normal.

In the current study, we found that both the AULCSF and Cutoff SF were significantly reduced in young patients with high myopia and normal best-corrected vision. The results are consistent with previous studies (10, 34). Importantly, we found weak correlations between CSF and chorioretinal thickness and vascular density of eyes in high myopia. A multiple regression analysis showed that reduced ORT and DVD were related to the CSF loss, especially ORT of the parafoveal and perifoveal. This is consistent with McAnany et al. (35) who found that the CSF was significantly correlated with outer retina thickness in early-stage diabetic retinopathy. So for high myopia, CSF may be a better endpoint than visual acuity for assessing and monitoring its occurrence and development.

The CSF can be affected by the function of photoreceptor cells (36). Campbell and Robson (36) suggested that contrast sensitivity in low spatial frequencies may be related to the function of Y-cell channels in peripheral retina, and contrast sensitivity in high spatial frequencies may be related to the function of X-cell channels in central vision. Under well controlled conditions, CSF has been used to evaluate retinal function in many studies (12–16). For example, Hoffmann et al. (12) measured visual functions and morphologic parameters of the retina in patients with age-related macular degeneration (AMD) and found that among all the measures of visual function, the CSF correlated best with anatomic features of the retina. As previous studies have shown, damages in the retinal and choroidal vascular support, such as vascular bed disease, can lead to severe visual impairment (37). We suggest that changes in retina and choroidal microcirculation may be an early manifestation of myopia-related diseases (38, 39), so when the outer layer of the retina atrophies and becomes thinner, CSF decreases. Moreover, our research found that the AULCSF was correlated with measures of parafoveal and perifoveal structures but not foveal structures. We speculate that one explanation could be that the foveal region is mainly an avascular zone, so it is more difficult to detect any differences at the fovea. On the other hand, the peripheral retina is possibly affected earlier than foveal retina in high myopia, and functional vision might be affected earlier than the microstructure of the fovea. Consistent with this view, Liou and Chiu (10) and Ang et al. (34) reported that in patients with high myopia, CSF was altered earlier than the foveal structure.

To our best knowledge, this is the first study to investigate the relationship between the CSF and fundus microstructure in subjects with simple early-stage high myopia. The study has some limitations. First, many factors may affect the CSF of patients with high myopia, such as high-order aberrations and the optical magnification of spectacles on objects. These factors were not considered in this experiment. Second, this is a cross-sectional study. Future longitudinal research analyzing the sequence of changes in the choroid and retinal structure and visual function is necessary to clarify the mechanisms underlying visual function damage in high myopia. Third, the form of optical correction could affect the observed CSF. In our experiment, refractive errors were corrected with spectacles. Liou and Chiu (10) found that, for high myopia, contact lens correction could reduce optical defocus and improve contrast sensitivity function in high spatial frequencies compared to spectacles. However, Ehsaei et al. did not find any significant difference in central and peripheral visual performance of myopic subjects under contact lens and spectacle lens corrections, even with the consideration of spectacle magnification (40). Moreover, in severe myopia, Liou and Chiu also found that the reduced contrast sensitivity could not be fully compensated by contact lens correction. In this study, we found that, in patients with simple high myopia, contrast sensitivity was correlated with measures of chorioretinal structure and vasculature. Although we can’t completely rule out contributions from imperfect optical correction, the reduced CSF in sHM observed here could not be fully explained by the remaining optical errors and is probably due to the structural changes in the retina.

## Conclusion

In conclusion, compared to low to moderate myopic eyes, patients with simple high myopia have thinner retinal and choroidal thickness, lower retinal vascular density, and reduced contrast sensitivity. In addition, their contrast sensitivity tested with corrected-to-normal visual acuity was correlated with measures of chorioretinal structure and vasculature. The results suggest that the CSF is a sensitive functional endpoint in simple early-stage high myopia.

## Data Availability Statement

The raw data supporting the conclusions of this article will be made available by the authors, without undue reservation.

## Ethics Statement

The study protocol adhered to the tenets of the Declaration of Helsinki and was approved by the Human subjects’ Research Review Board of the Eye Hospital of Wenzhou Medical University. The patients/participants provided their written informed consent to participate in this study.

## Author Contributions

XL and YW collected and analyzed the data and wrote the first draft of the manuscript. XF, FZ, JH, HY, QW, and MZ collected the data. FH and LL analyzed and interpreted the data. ZL, FL, and XM designed and supervised the study and wrote the manuscript. All authors contributed to the revision of the manuscript.

## Conflict of Interest

LL and ZL own intellectual property rights on the qCSF technology and have equity interest in Adaptive Sensory Technology, Inc. LL holds employment at Adaptive Sensory Technology, Inc. The remaining authors declare that the research was conducted in the absence of any commercial or financial relationships that could be construed as a potential conflict of interest.

## Publisher’s Note

All claims expressed in this article are solely those of the authors and do not necessarily represent those of their affiliated organizations, or those of the publisher, the editors and the reviewers. Any product that may be evaluated in this article, or claim that may be made by its manufacturer, is not guaranteed or endorsed by the publisher.

## Abbreviations

CSF: contrast sensitivity function
qCSF: quick contrast sensitivity function
AULCSF: the area under log CSF
Cutoff SF: the cutoff spatial frequency
AL: axial length
BCVA: best corrected visual acuity
sHM: simple high myopia
SVD: retinal superficial vascular density
DVD: retinal deep vascular density
IRT: inner retinal thickness
ORT: outer retinal thickness
ChT: choroidal thickness.
